# Robotic-Assisted Lobectomy Following Induction Chemoimmunotherapy Achieves Complete Pathologic Response in Stage IIIA Lung Adenocarcinoma: A Case Report

**DOI:** 10.7759/cureus.92285

**Published:** 2025-09-14

**Authors:** Kendall Howard, Ray Chihara, Warren C Naselsky, Min P Kim

**Affiliations:** 1 Surgery, Houston Methodist Hospital, Houston, USA

**Keywords:** induction chemoimmunotherapy, kras mutation, lung cancer, pathologic complete response, pathologic complete response (pcr), robotic lobectomy, stage iiia, t4 lung cancer

## Abstract

Novel therapies for clinical stage IIIA lung cancer are changing the outcomes in advanced clinical stage lung cancer. A 77-year-old female patient diagnosed with clinical T4 or stage IIIA right lower lobe adenocarcinoma with a KRAS mutation. She received neoadjuvant chemoimmunotherapy for an 8 cm tumor, which showed a moderate response on imaging. The patient underwent robotic-assisted thoracoscopic right lower lobectomy and mediastinal lymph node dissection. The final pathology showed a complete response. Novel induction chemoimmunotherapy provides an opportunity to completely eradicate cancer and provides surgical therapy for patients with advanced lung cancer.

## Introduction

Lung cancer remains the leading cause of cancer-related deaths worldwide and in the United States, with 225,650 new cases and 124,730 deaths estimated by 2025 [1]. The overall decrease in prevalence can be attributed to the decrease in the number of smokers and therapeutic advancements. However, there are high rates of recurrence and death; an estimated 64% of patients with stage IIIA disease will eventually die within five years [2]. Surgical resection is the mainstay of treatment for early-stage disease; however, multimodal therapy has been the mainstay for advanced disease, especially stage IIIA lung cancer [2,3].

Traditionally, induction therapy has been delivered with platinum-based chemotherapy and has shown only a marginal decrease in the five-year survival rate of 5% compared to surgery alone [4]. Recently, a randomized controlled trial for patients with resectable stage IIIA or IIIB disease treated with induction chemoimmunotherapy followed by surgery showed a significant increase in the pathologic complete response rate and two-year overall survival compared to controls [5]. Here, we present a case of large T4N0M0 or stage IIIA right lower lobe non-small cell lung cancer (NSCLC) treated with neoadjuvant chemoimmunotherapy followed by robotic lobectomy.

## Case presentation

A 77-year-old female patient with a smoking history of 17 pack-years who had quit smoking seven years ago presented with an 8 cm lung mass. She was first noted to have a small right lower lobe 2 cm ground glass opacity when undergoing evaluation for interstitial lung disease four years prior to presentation. The patient was recommended a pulmonologist for biopsy, but was lost to follow-up until she presented to the emergency department with worsening of persistent dry cough with intermittent hemoptysis after recovering from COVID-19 infection.

**Figure 1:**
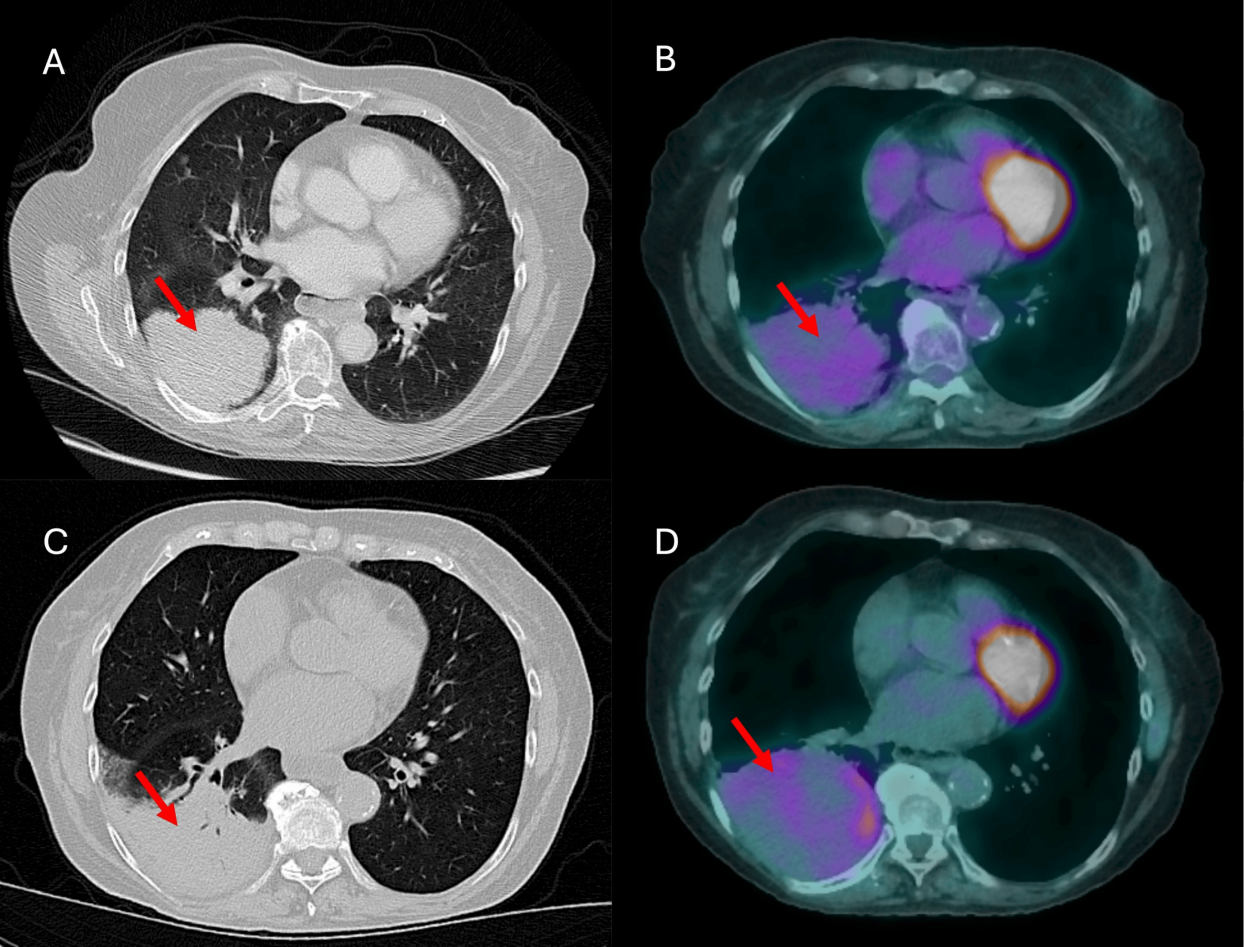
CT and PET/CT imaging (A) CT of the right lower lobe lung cancer (red arrow) and (B) PET/CT of the cancer before induction of chemoimmunotherapy.  (C) CT and (D) PET/CT images of the right lower lobe cancer after chemoimmunotherapy.

A chest computed tomography (CT) scan showed an 8 cm mass in the right lower lobe (Figure 1A). The patient underwent transbronchial biopsy, which was positive for invasive adenocarcinoma with KRAS mutation p.G12V, with no programmed death-ligand 1 (PDL1) tumor cell staining and <1% staining of tumor-associated inflammatory cells. Subsequent nodal staging with endobronchial ultrasound-guided biopsy of stations 4 L, 7, and 11R was negative for metastasis. The patient underwent positron emission tomography(PET)/CT (Figure 1B) and brain magnetic resonance imaging (MRI), which did not show any signs of metastatic disease. The patient was clinically diagnosed with T4N0M0 or stage IIIA lung cancer. 

The patient was discussed at the thoracic tumor board. Since the patient did not have driver mutations such as epidermal growth factor receptor (EGFR), anaplastic lymphoma kinase (ALK), or ROS proto-oncogene 1, receptor tyrosine kinase (ROS1), and she had a good performance status, she was recommended to undergo induction chemoimmunotherapy followed by surgery. The patient completed three cycles of neoadjuvant chemotherapy with cisplatin, pemetrexed, and nivolumab, with stable disease on re-imaging with PET/CT (Figures 1C, 1D). The patient had good pulmonary function and adequate cardiovascular reserve to undergo surgery; however, she needed to recover from induction therapy. The patient was enrolled in a pre-habilitation program where she established care with a physical therapist and nutritionist prior to surgery. 

**Figure 2:**
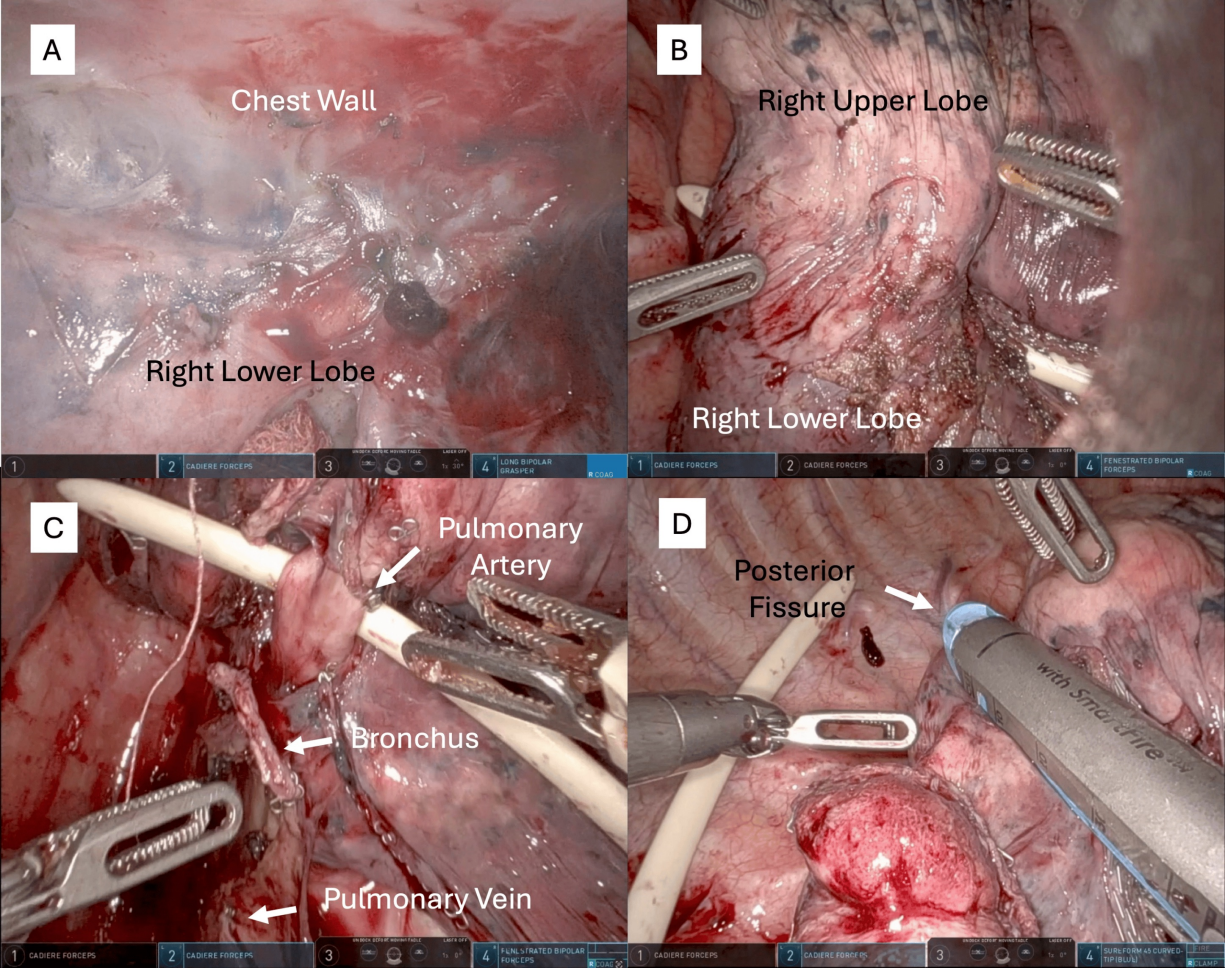
Intraoperative images (A) Dense adhesion between the right lower lobe and the chest wall without signs of cancer invasion into the chest wall. Adhesion was observed between the right lower lobe and the right upper lobe. (B) An endoleader was used to perform en bloc wedge resection of the right upper lobe.  (C) Division of the basilar segmental artery of the right lower lobe with the aid of the endoleader with divided right lower lobe bronchus and inferior pulmonary vein. (D) Division of incomplete posterior fissure using robotic stapler.

The patient underwent robotic-assisted thoracoscopic right lower lobe lobectomy with mediastinal lymph node dissection. Upon gaining access to the thoracic cavity, dense adhesions were observed between the tumor and chest wall (Figure 2A). A biopsy of the area showed no signs of malignancy. The tumor in the right lower lobe also adhered to the right upper lobe (Figure 2B), rendering hilar dissection challenging. En bloc resection of the right upper lobe wedge, along with the mass, was performed to ensure satisfactory margins. Robotic visualization and instrument articulation of the pulmonary vein, right lower lobe bronchus, and superior segmental and basilar pulmonary arteries were sequentially divided using a robotic stapler (Figure 2C). Dissection was aided by an endoleader to guide the stapler across the bronchovascular structures. Finally, the fissure was divided to complete the lobectomy (Figure 2D, Video 1).

**Video 1:** Robotic-assisted lobectomy following induction chemoimmunotherapy for stage IIIA lung cancer

The patient recovered well from the surgery, her chest tube was removed, and she was discharged on postoperative day 2. She was discharged on a multimodal pain control regimen (acetaminophen 1 g every eight hours (q8H), gabapentin 300 mg q8H, methocarbamol 500 mg q8H, and tramadol, as needed). The patient progressed as expected during her two- and six-week follow-up visits. Pathological examination of the specimen revealed a pathological complete response. Follow-up surveillance imaging performed 12 months later showed no evidence of disease recurrence.

## Discussion

The incorporation of chemoimmunotherapy and robotic surgery has changed the way we think about the treatment of advanced lung cancer. A phase II trial, the Neo-Adjuvant Immunotherapy with Nivolumab for NSCLC Patients (NADIM II), compared induction chemoimmunotherapy followed by surgery and immunotherapy to induction chemotherapy followed by surgery for resectable stage IIIA and IIIB NSCLC and showed that more patients were able to undergo surgery after chemoimmunotherapy (93% vs. 69%), with significantly higher rates of pathologic complete response (37% vs. 7%); patients who received induction chemoimmunotherapy had an overall survival of 67.2% at two years, which was significantly better than that of the control group (40.9 %) [5]. A subsequent landmark phase 3 trial, CheckMate 816, showed that patients with stage IIIA NSCLC who received nivolumab with chemotherapy prior to surgery had a significant improvement in the event-free survival of 31.6 months compared to induction chemotherapy alone (20.8 months, HR 0.63) [6]. In addition, patients who received chemotherapy had a pathologic complete response rate of 24% compared to those who received chemotherapy alone (2.2%). This has led to the use of this induction regimen in patients with advanced lung cancer.

Prior to starting advanced-stage NSCLC treatment with induction chemoimmunotherapy, the molecular profile of the tumor must be checked. Patients with EGFR, ALK, or ROS1 mutations in tumors have very low response rates to induction immunotherapy [7]. However, similar to our patient, patients with KRAS mutations respond to chemoimmunotherapy [8]. Thus, induction chemoimmunotherapy should not be administered to patients with EGFR, ALK, or ROS1 mutations.

We obtained imaging after chemoimmunotherapy to ensure that there were no signs of progression after therapy. In the CheckMate 816 study, 10.6% of patients did not undergo surgery due to disease progression [6]. Although there was no progression on imaging, a large tumor was still present at the primary site. CT after chemoimmunotherapy can show a response, but cannot reliably show a pathologic complete response due to its limitations in distinguishing fibrosis from necrosis. In CheckMate 816, CT complete response was rare [6].

The best surgical option after induction chemoradiation therapy is robotic technology. It allows for careful dissection of the hilar structures using a minimally invasive method and improves the number of lymph nodes dissected compared to non-robotic minimally invasive methods [9]. In addition, robotic surgery has transformed patient outcomes with decreased pain, shorter length of stay, and fewer complications than open and video-assisted thoracoscopic (VATS) techniques, which allow for faster recovery of patients [10,11]. In addition, the use of the Enhanced Recovery After Surgery (ERAS) protocol with prehabilitation and protocol-driven care after surgery significantly improves outcomes [12]. 

## Conclusions

This case highlights the potential of novel induction chemoimmunotherapy to achieve pathologic complete response in patients with advanced clinical stage lung cancer, underscoring its role in enabling curative resection. The integration of robotic-assisted surgical technology further enhances the precision and safety of these complex procedures, offering an opportunity to improve outcomes in appropriately selected patients.
